# Engineering of Cytolethal Distending Toxin B by Its Reducing Immunogenicity and Maintaining Stability as a New Drug Candidate for Tumor Therapy; an In Silico Study

**DOI:** 10.3390/toxins13110785

**Published:** 2021-11-05

**Authors:** Maryam Keshtvarz, Mahdieh Mahboobi, Marek Kieliszek, Antoni Miecznikowski, Hamid Sedighian, Milad Rezaei, Mohammad Ali Haghighi, Zahra Zareh, Ehsan Rezaei

**Affiliations:** 1 Department of Microbiology and Parasitology, Faculty of Medicine, The Persian Gulf Tropical and Infectious Diseases Research Center, Bushehr University of Medical Sciences, Bushehr 7514633341, Iran; mkeshtvarz@gmail.com (M.K.); mahaghighy@gmail.com (M.A.H.); 213zaree@gmail.com (Z.Z.); 2 Applied Microbiology Research Center, Systems Biology and Poisonings Institute, Baqiyatallah University of Medical Sciences, Tehran 1435916471, Iran; mahdiehmahboubi@gmail.com (M.M.); osedighian@gmail.com (H.S.); 3 Department of Food Biotechnology and Microbiology, Institute of Food Sciences, Warsaw University of Life Sciences-SGGW, Nowoursynowska 159 C, 02-776 Warszawa, Poland; marek_kieliszek@sggw.edu.pl; 4 Prof. Waclaw Dabrowski Institue of Agricultural and Food Biotechnology, State Research Institute, Rakowieka 36, 02-532 Warszawa, Poland; antoni.miecznikowski@ibprs.pl; 5 Biology Department, Sciences Faculty, Brujerd Branch, Islamic Azad University, Brujerd 6915136111, Iran; mi.rezaei@yahoo.com; 6 Molecular Biology Research Center, Systems Biology and Poisonings Institute, Baqiyatallah University of Medical Sciences, Tehran 1435916471, Iran

**Keywords:** cytolethal distending toxin, immunogenicity, stability, tumor therapy, mutation

## Abstract

The cytolethal distending toxin (CDT), *Haemophilus ducreyi*, is one of the bacterial toxins that have recently been considered for targeted therapies, especially in cancer therapies. CDT is an A-B2 exotoxin. Its catalytic subunit (CdtB) is capable of inducing DNA double strand breaks, cell cycle arrest and apoptosis in host eukaryotic cells. The sequence alignment indicates that the CdtB is structurally homologyr to phosphatases and deoxyribonucleases I (DNase I). Recently, it has been found that CdtB toxicity is mainly related to its nuclease activity. The immunogenicity of CDT can reduce its effectiveness in targeted therapies. However, the toxin can be very useful if its immunogenicity is significantly reduced. Detecting hotspot ectopic residues by computational servers and then mutating them to eliminate B-cell epitopes is a promising approach to reduce the immunogenicity of foreign protein-based therapeutics. By the mentioned method, in this study, we try to reduce the immunogenicity of the CdtB- protein sequence. This study initially screened residue of the CdtB is B-cell epitopes both linearly and conformationally. By overlapping the B-cell epitopes with the excluded conserve residues, and active and enzymatic sites, four residues were allowed to be mutated. There were two mutein options that show reduced antigenicity probability. Option one was N19F, G74I, and S161F with a VaxiJen score of 0.45 and the immune epitope database (IEDB) score of 1.80, and option two was N19F, G74I, and S161W with a VaxiJen score of 0.45 and IEDB score of 1.88. The 3D structure of the proposed sequences was evaluated and refined. The structural stability of native and mutant proteins was accessed through molecular dynamic simulation. The results showed that the mutations in the mutants caused no considerable changes in their structural stability. However, mutant 1 reveals more thermodynamic stability during the simulation. The applied approaches in this study can be used as rough guidelines for finding hot spot immunogen regions in the therapeutic proteins. Our results provide a new version of CdtB that, due to reduced immunogenicity and increased stability, can be used in toxin-based drugs such as immunotoxins.

## 1. Introduction

Cancer is one of the leading causes of death worldwide. One of the causes of cancer is the disruption of the apoptotic pathway by the overexpression of anti-apoptotic proteins and under-expression of pro-apoptotic proteins, which results in intrinsic resistance to the most common anti-cancer treatments [1,2,3]. Despite the latest technological advances in conventional therapies, such as chemotherapy and radiotherapy, these techniques still have certain limitations due to the severe side-effects on normal tissue [4]. On the other hand, it has recently been shown that some bacterial toxins have the ability to kill cancer cells or reduce the cellular processes that control proliferation, apoptosis, and differentiation [5,6,7,8,9,10]. For example, the Cytolethal distending toxin (CDT) can induce apoptosis in the majority of human leukemic T cells (MOLT-4) through the caspase-dependent classical pathway [11,12]. CDT is produced by some Gram-negative bacteria such as *Aggregatibacter actinomycetemcomitans*, *Haemophilus ducreyi*, *Helicobacter hepaticus, Campylobacter jejuni*, *Escherichia coli*, and others [13,14,15]. CDT is composed of three subunits, A, B, and C, which form an AB_2_ heterotrimer toxin [16]. CdtA and CdtC are necessary for receptor binding and translocation of the active subunit (CdtB) into the host cells [17]. CdtB is an catalytic subunit capable of inducing double strand breaks (DSBs) and single strand breaks (SSBs), G2/M cell cycle arrest, and eventually apoptotic death [18,19]. Compared to the CdtA and CdtC sequences, which show a higher degree of variability, the CdtB sequence is more conserved [20]. The CdtB exhibits phosphatidylinositol-3,4,5-triphosphate (PIP3) phosphatase activity and induces phosphoinositide 3-kinase (PI-3K) signaling blockade [21]. PI3K signaling complex plays a key regulatory role in many cellular processes, such as cell survival, proliferation and differentiation [18,22]. In addition to phosphatase activity, CdtB has a structural homology to deoxyribonuclease I (DNase I) that is dependent on several key residues; so the removal of any of these residues can reduce the nuclease activity of the CdtB [16]. Recently, Pons et al. reported that CDT toxicity is mainly dependent on CdtB nuclease activity, while phosphatase activity may be involved in CdtB intracellular trafficking [18].

Currently, cancer therapy using all subunits, or only the enzymatic subunit of CDT in combination with some targeted moieties, has received much interest. Bahan et al. reported that conjugation of the enzymatic subunit of *Haemophilus ducreyi* (HdCDT), CdtB, with the N-terminal 255 amino acids of *Bacillus anthracis* toxin lethal factor (LFn), has great potential as an anticancer treatment [13]. Additionally, Yu-An Chen et al. used delivered nanoparticles based on hyaluronic acid for delivered CdtB to prostate cancer. They show that this composition has activity similar to CDT whole toxin, but with the difference that the CdtB can be delivered specifically to cancer cells and enhance the effect of ionizing radiation in radio-resistant prostate cancer cells [23]. In another work by Vafadar and colleagues, the new immune-toxin based on scFv-CdtB were designed and evaluated against breast cancer by in silico methods [24].

The main problem of using bacterial toxins as anti-cancer drugs is that many of them are very immunogenic in humans with normal immune systems [25]. So, when these toxins are used especially against solid tumors, they develop neutralizing antibodies and prevent additional treatment cycles. In addition, anti-therapeutic antibodies can rarely trigger life-threatening immune responses [26]. Immune responses to protein therapeutics can be divided into two categories, depending on whether the protein is foreign or autologous [27]. Bacterial toxins, such as *Haemophilus ducreyi* Cdt (Hd Cdt), are foreign proteins that can activate the classical immune system, including T cells, B cells, dendritic cells (DCs), monocytes, and macrophages [28,29]. While humanized antibodies are autologous proteins, which may engender complicated immune responses, including immune tolerance [30].

Preclinical immunogenicity studies have been limited to monitoring antibody production in rodents and non-human primates. The immunogenicity of bacterial toxins in animals is often predictive for humans [31]. For example, immunization with individual CdtA, CdtB and CdtC proteins of *Haemophilus ducreyi* is able to induce high antibody titer with toxin-neutralizing activities [32]. Several approaches have been proposed to reduce the immunogenicity of therapeutic proteins, the most important of which include site-specific modification of the protein with polyethylene glycol, which masks the immunogenic epitopes, or the identification and elimination of B- and T-cell epitopes that are recognized by the immune system [33].

The toxins engineering toward hiding immunogenic epitopes from the immune system has recently received a great deal of attention. In earlier studies, bioinformatics approaches have helped researchers to find the key immunogenic epitopes and its engineering [34]. Both linear and conformational B cell epitopes are important to induce B cells, resulting in a high immunogenic reaction [35,36].

So far, CdtB has not been manipulated to reduce immunogenicity while this component has high potential as an anticancer drug. Here, we aimed to identify the linear and conformational B cell epitopes with the best presence computational tools. By considering catalytic sites and conserved regions of the protein, we recognized the hotspot points and changed with the ideal residues to design a new CdtB to reduce immunogenicity and retain stability.

## 2. Results

### 2.1. Retrieving Native CdtB Sequence

**Figure 1:**
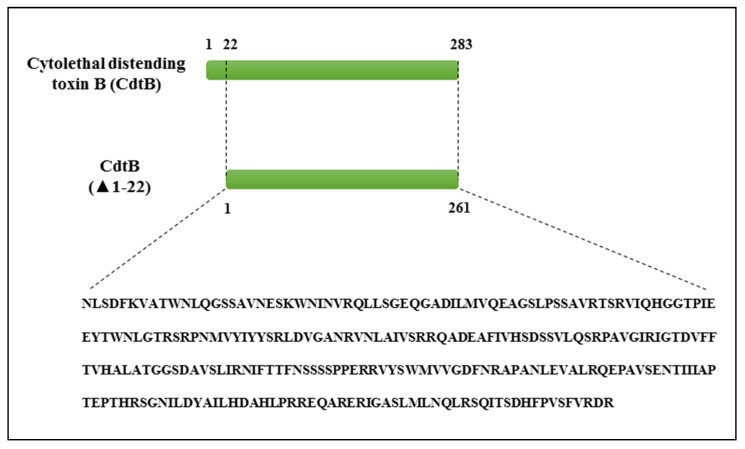
The sequence of CdtB without signal peptide.

The CdtB of Haemophilus ducreyi (UniProt accession number: O06523) consists of 283 amino acids, of which the first 22 amino acids are the signal peptide, which was omitted in this study. The remaining amino acids (261aa) were then examined for antigenic properties and the presence of antigen epitopes (Figure 1).

### 2.2. Antigenic Properties of the CdtB Toxin

VaxiJen [37] is a server for alignment independent prediction of protective antigens of tumour, bacterial, and viral origin. The threshold score of VaxiJen server for bacterial species is 0.4, the predicted antigenicity probability of CdtB was 0.53 which was higher than the threshold. Similar to the experimental results [32,38], this result shows that the toxin has antigenic properties.

### 2.3. B-Cell Epitope Prediction

**Figure 2:**
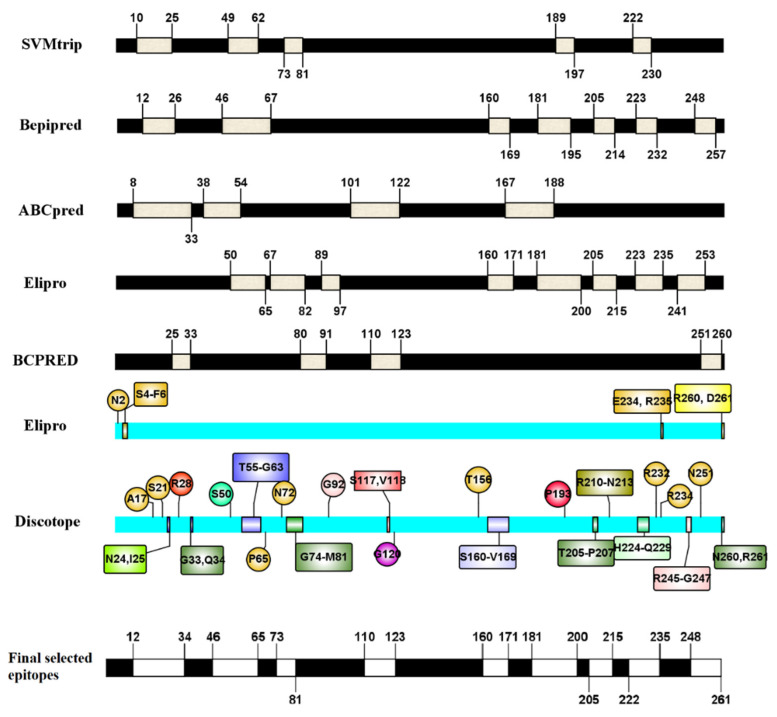
The linear B cell epitopes identified by SVMTriP, BepiPred2.0, ABCpred, Elipro and BCPREDS, and also conformational B cell epitopes identified by DiscoTope 2.0 and Elipro servers are shown. Final selected epitopes are introduced by at least 3 servers.

Accurate analysis of linear epitopes is difficult because these tools are primarily designed to predict fixed-length peptides, usually between 9 and 20 amino acid residues [32,39]. The lowest range size to determine the linear B-cell epitope was considered as 9 amino acids [40]. All predicted epitopes larger than 9 aa that predicted using bepipred and elipro servers were selected. Epitopes with various lengths (10aa, 12aa, 14aa, 16aa, 18aa, 20aa, and 22aa) were predicted using ABCpred [41], SVMtrip [42] and BCPREDS servers. Five high-scored epitopes of various lengths at ABCpred were selected and among them, various lengths of epitopes that overlapped at least three times were determined as final ABCpred epitopes. Concerning SVMtrip and BCPREDS servers, firstly, various lengths of epitopes with score 0.8 or higher were identified and then the overlapping regions of them were selected. DiscoTope 2.0 and Elipro servers were used to predict conformational B cell epitopes. Fifty-four residues were identified as B cell epitopes using DiscoTope 2.0 and eight residues were introduced as non-linear or conformational epitopes by Elipro. Finally, identified epitopes by at least three algorithms (linear and conformational servers) were selected for further evaluation. The identified epitopes by any algorithm and the final selected epitopes are shown in Figure 2.

### 2.4. Conserved and Functional Residues Determination

**Figure 3:**
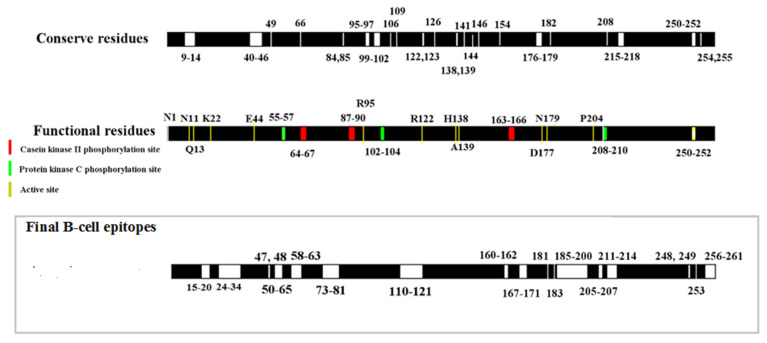
Graphical illustration of the conserved region, functional residues and final B-cell epitopes.

Structural similarities between the active sites of CdtB with the other PDB protein structures were detected by ProBiS database. The results showed that the CdtB contains 14 residues as functional binding sites (active sites) that are very much similar to DNAS1_HUMAN (pdb: 4AWN) with Z score 2.27. The enzymatic sites and conserved regions of CdtB were determined by PredictProtein and ProBis tools. Overall, some residues were known as key residues for CdtB activity including 50 residues as conserved regions, 14 residues as active sites, 9 residues as protein kinase C phosphorylation sites and 12 residues as Casein kinase II phosphorylation. As shown in Figure 3, the mutable residues were identified by combining functional residues and final B-cell epitopes.

### 2.5. Hotspot Regions Identification

Hot spots are usually defined as those residues for which ∆∆G ≥ 2 kcal/mol [43]. The changeable residues in final B- cell epitopes mutated to the 19 other naturally occurring amino acids, using I-Mutant 2.0 to determine specific amino acids with the highest positive ΔΔG or more stability. Finally, as shown in Table 1, residues were selected for mutation that had the highest IEDB score, hydrophilicity, and surface accessibility. IEDB contains information related to antibodies and T cells across an expansive scope of research fields (infectious diseases, allergy, autoimmunity, and transplantation) [44]. It is noteworthy that some residues could mutate to more than one amino acid with the highest positive ΔΔG. Therefore, using VaxiJen, amino acids were selected that had the lowest antigenic scores. Then, according to Table 1, all selected residues were replaced with appropriate amino acids in their specific B cell epitopes.

As shown in Table 1, N19, G74, S161, and S162 residues were known as hotspot residues that could mutate to amino acids that significantly reduced antigenic and IEDB scores of highly antigenic B cell epitopes. Each residue was replaced by a suitable amino acid, except for S161 residue that could be replaced with W or F amino acid (Table 2. It is noteworthy that a single mutation on S161 was able to completely remove the antigenicity of the S**S**SSPPERRVYS (160–171) epitope, so mutation on S162 was not performed and excluded from the study. In summary, two mutation options were proposed. Option one was N19F, G74I, and S161F with a VaxiJen score of 0.45 and IEDB score of 1.80. Option two was N19F, G74I, and S161W with a VaxiJen score of 0.45 and IEDB score of 1.88 (Table 3).

### 2.6. Tertiary Structure Prediction, Refinement and Validation

**Figure 4:**
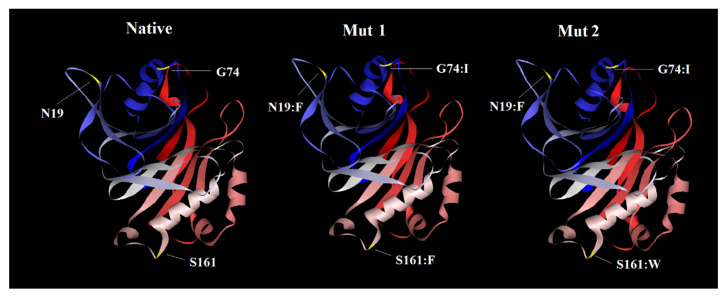
Predicted tertiary structure of the native protein versus mut-1 and mut-2. The mutations are N19:F, G74:I, S161:F in mut-1 and N19:F, G74:I, S161:W mut-2 and their points are shown in the fig.

**Figure 5:**
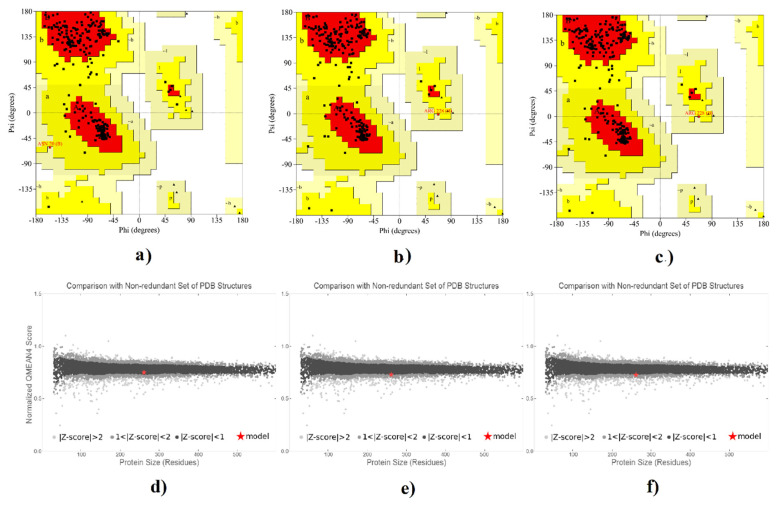
Analysis of 3D models of the native protein versus mut-1 and mut-2. Ramachandran plot of (**a**) the native structure, (**b**) mut-1 and (**c**) mut-2. Quality comparison of (**d**) the native structure (**e**) mut-1 and (**f**) mut-2.

For 3D structure prediction of CdtB, Mut 1 and Mut 2, the homology modeling procedure was applied using the SWISS MODEL server. Among the produced templates, the 1sr4 template, due to 100% sequence identity with the native model, was selected and applied for all structures. In comparison with the 1sr4 template, Mut 1 and Mut 2 showed the proper protein folding (Figure 4). The z-scores results validating our mutant models are within the range of scores typically found for native proteins of similar size. Final structure’s evaluation was performed, followed by the protein structure refinement using the 3D refine program where the Ramachandran plot statistics showed a good quality for all three structures. Ramachandran plot displayed 89.2% and 10.3% of residues in native model located in the most favored and additional allowed regions, respectively. Regarding Mut 1 and Mut 2, Ramachandran plot statistics indicated 89.7% and 90.1% of their residues clustered in the most favored regions, respectively, and also 9.9% and 9.4% of their residues clustered in the additional allowed regions (Figure 5). When over 90% of residues of a protein are placed in the most favored regions, protein models have good quality [45]. So, it seems all of the structures had proper φ and ψ torsion angles and were located in the low energy areas. The main reasons for placing the mutant protein residues in the most favored or allowed region, near 90%, is that there are no dramatic changes in mutants compared to the native protein. The 3D models were analysed by ERRAT for evaluating the overall quality factor and by verifying 3D for compatibility validation of their atomic model (3D) with its own amino acid sequence (1D). Verifed 3D indicates the acceptable values of 3D/1D compatibility for the mutants compared to the native model, while overall quality values for the mutants are less than native (Table 4).

### 2.7. Molecular Dynamics Simulation Results

Molecular dynamics simulation can give us important data about individual atom movement. This data is usable for prediction of protein folding while they cannot be easily obtained by experimental assessments. The MD simulation results were applied to compare the thermodynamic stability of the models.

**Figure 6:**
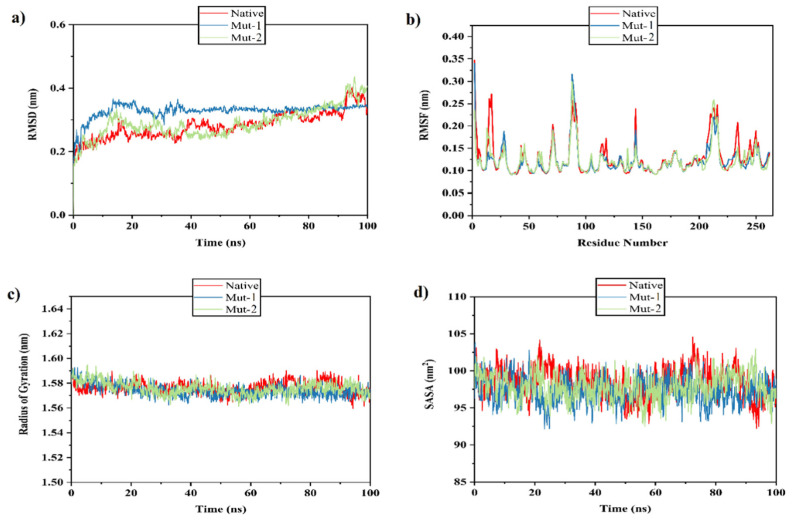
Analysis with RMSD, RMSF, Rg and SASA. (**a**) Plot of time vs. RMSD trajectory of native, mut-1 and mut-2 protein models at 100 ns. (**b**) RMSF plot for native, mut-1 and mut-2. (**c**) Time evolution of the radius of gyration (Rg) value for native, mut-1 and mut-2. (**d**) Solvent accessible surface area (SASA) plot for native, mut-1 and mut-2. Native [47], mut-1 (blue) mut-2 (green).

As a standard factor for assessments of the structural stability, the RMSD factor on the backbone atoms of protein models was computed. RMSD profile showed that the system was equilibrated from the 15th ns until the end of the simulation. The model 2 displays the same pattern with the native model but the model 1 shows a steady state and a considerable thermostability through the simulation period (Figure 6a).

The RMSF factor shows the displacement of a specific atom, or group of atoms, compared to the reference structure [46]. The RMSF value of the modeled structures was investigated to define the effect of mutations in the dynamic behavior of the adjacent residues. So, as seen in Figure 6b, the RMSF value has been assessed in 3 points in which the mutations have occurred. In point 1, more fluctuations are seen in the native residues relative to the mutants. Additionally, the mut-2 residues exhibited more movements, relative to the mut-1 residues in this position. Interestingly, the increase in flexibility did not cause more structural stability in the modeled structures and it seems they showed no significant difference in the fluctuations of their residues at the points 2, 3 and even other residues.

The radius of gyration (Rg) of a protein is the root mean square distance of each atom from the core of the protein. However, when the Rg value is lower, the folding of the protein over time is better. As shown in Figure 6c, all modeled structures displayed a steady state in the Rg value of about 1.58 nm, showing a proper structural folding throughout the simulation period.

We applied the solvent- accessible surface area (SASA) to find the behavior of the hydrophilic and hydrophobic residues of the native, mut-1 and mut-2 models. The results revealed that the amino acid residues of the mut-1 and mut-2 structures have a similar or lower SASA value as compared to the native structure, and they maintained the solvent accessibility during a simulation time of 100 ns (Figure 6d).

**Figure 7:**
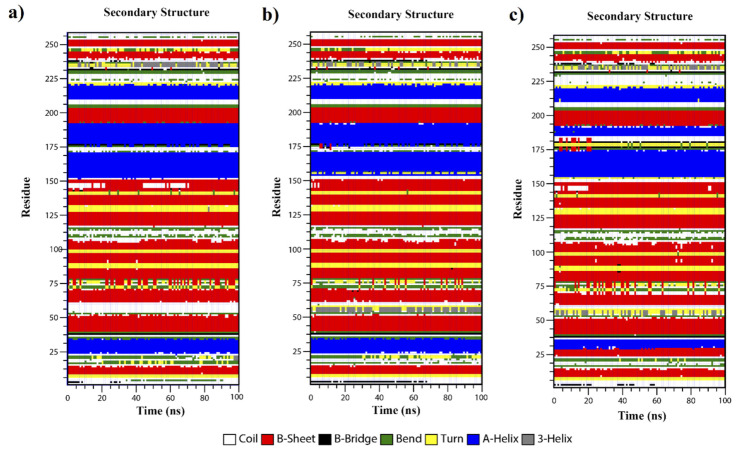
Secondary structure graph fluctuations as a function of time from 0 to 100 ns for the native (**a**), mut-1 (**b**) and mut-2 (**c**) models.

We used DSSP to compute the secondary structure of produced frames during simulation for all models. As seen in Figure 7, the secondary structure of the mut-1 for the first 30 residues is completely the same with the native model, while shift from alpha-helix to beta-sheet (about residue 25) is observed in the mut-2. The residues in the range of 150 to 190 also formed stable β-sheet structures in mut-1, which was not shown about mut-2. This part of the mut-2 showed the changes from alpha-helix to the coil and turn that forms a high level of instability in the mut-2. These observations were in agreement with our previous results such as RMSD and RMSF, which revealed the more stability of mut-1 during simulation. The minimal differences between mutein 1 and 2 were shown in Table 5.

## 3. Discussion

Stability, toxicity, and immunogenicity are key factors that should be considered in the selection of anti-cancer toxins. Yamada et al. found that HdCdtB in comparison with *Actinomyces actinomycetemcomitans* (AaCdtB), can maintain both its stability and toxicity in the presence of a buffered solution containing 10% sucrose [48]. On the other hand, Wising et al. indicated that the therapeutic effects of the CdtB can be reduced due to having many antigenic regions resulting in development of the naturalizing antibodies anti-cancer toxins [32].

In the present study, to reduce immunogenicity, we designed the lower antigenic type of CdtB by identification and modifications of its B cell epitopes using in silico approaches. The methods provide a preliminary study for mutagenesis and identify which amino acid residue in B cell-epitope should be mutated to reduce antigenicity while still maintaining its stability and activity compared to the native protein.

The antigenicity of the CdtB sequence was first analyzed using Vaxijen software [37]. The software predicted that this sequence was highly antigenic, consistent with the experimental results [32]. B-cell epitopes are antigenic regions on the surface of a protein that can control most of the immune response. Therefore, elimination of these epitopes leads to antibody-mediated neutralization and ultimately suppression of the immune response [49,50]. As we know, B cell epitopes can be categorized into two types: linear (continuous) and conformational (discontinuous) epitopes [51]. Various tools have been suggested to identify linear, such as ABCpred [41], BepiPred [52], SVMtrip [42] and BCPred [53]. These tools are based on several items such as highest accuracy, specificity, positive predictive and Matthews correlation coefficient [29,39]. The majority of antigenic epitopes recognized by B cells are conformational [54]. So, any mutation that can alter this special conformation may reduce the antigenicity [55,56,57]. The conformational epitopes could be predicted by DiscoTope 2.0 [35] and Elipro [58] based on (both solvent-accessibility-based properties and epitope log odds) and (grouping surface residues under their properties index), respectively. Overall, this study based on the mentioned tools showed that most B-cell epitopes of the cdtB are conformational.

Some residues are critically important for the protein function and structural integrity, including active site residues, conserved residues, and protein kinase C phosphorylation and Casein. PSI-BLAST sequence alignment reveals that the CdtB protein has homology with the endonuclease/exonuclease/phosphatase proteins family [16]. Based on literatures and in silico analysis, the CdtB shares eleven conserved residues with the active sites of DNase I-nucleases, including N11, E44, R95, V96, R122, H138, D177, N179, D251, D216, and H252 (residue number has been written after deletion of the signal peptide with 23 residues) [21,59]. So any mutation or structural interaction in these residues can affect the nuclease activity of CdtB, which may be essential for the CDT-induced cell cycle arrest [59,60].

The crystal structure of CDT shows several regions of interaction between the 13-N terminal amino acid of CdtC and some active site residues of CdtB such as His 160; which were able to potentially reduce the catalytic activity of CdtB in the host cells [60,61]. Moreover, Elwel et al., by creating five mutations in the CdtB active site residues, showed that four of them can inhibit DNase activity and prevent induction of cell dilation or arrest of HeLa cells [59]. So, it is very important that these residues remain intact and the mutation does not occur in this area or near the active site [59]. In this study, to prevent any aberrant mutations in B cell epitopes that could reduce or inhibit the activity of the CdtB subunit, critical residues of this subunit were identified using ProBiS and Predicted Protein servers. Then, by removing critical residues from the nine final B-cell epitopes, mutable or changeable residues were identified. Finally, our selected points to mutation are in the proper distance of the mentioned active residues.

There are a few major residues that contribute to the formation of epitope hotspots (epitope cores) and based on their removal by point mutations, we can inhibit the binding of many antibodies associated with the epitope group; resulting in reducing the immunogenicity of the whole molecule [47,62]. It should also be noted that most of the epitope hotspots are polar and lack large hydrophobic residues [63]. Liu et al. identified the B cell-epitopes of PE38 in an immunotoxin against CD22 and removed large polar residues such as arginine. They introduced HA22-LR-LO10 immunotoxin in which domain II was deleted and seven-point mutations with substitution of alanine (very small non-polar residue) in domain III were entered. Results showed the immunogenicity of this immunotoxin remarkably was reduced while its antitumor activity was retained [64]. Moreover, Ramya et al. reported that substitution of small polar residues (such as D and E) with large non-polar residues (L and V) in three B-cell epitopes, led to reduction of L-Asparaginase immunogenicity [65]. Our study also indicated that substitution of small polar (N19 and S161) to large non-polar residues (F & W or F & F) and very small non-polar (G74) residues to large non-polar residues (I), in three hotspot regions of B-cell epitopes, LQGSSAV**N**ESKWNINVRQLLSGE (12-34), L**G**TRSRPNM (73-81) and S**S**SSPPERRVYS (160-171), can completely eliminate these antigenic epitopes, resulting in a reduction in the amount of B-cell level against CdtB. Therefore, two options with three-point mutations were designed, mutant 1 (N19F, G74I, and S161 W) and mutant 2 (N19F, G74I, and S161F). It is noteworthy that unlike N19, which was only in linear epitopes, G74 and S161 were also found in conformational epitopes.

The MD simulations results confirmed the stability of the 3D structure of the native and both mutein proteins. It seems that there is no considerable change in the stability of the mutant 1 versus native proteins. Interestingly, we found a small difference in the mutant 1, making it superior to the mutation 2. These advantages in the mutant 1 include the higher stable β-sheet structures, less fluctuations and movement, and finally a steady state and considerable thermostability through the simulation period. In a similar way, Tjoa et al. proposed two muteins for decreasing the immunogenicity of botulinum toxin type A so that option one was ΔE147, E510F, T1062F, ΔE1080, N1089M, ΔQ1090 and option two was ΔE147, E510F, T1062F, E1080W, N1089M, ΔQ1090. They used two phenylalanine residues in both of their muteins and ΔE1080 in the mutein 1 versus E1080W in the mutein 2 was the key factor that caused more flexibility in the mutein 2 [66]. However, the difference was minor, so it probably did not disturb the functional behaviors of the protein [67,68,69]. Despite that, helix to coil transition in the mutant 2 may cause the alteration in function of protein, but only experimental analysis can approve or reject this hypothesis. According to our results, both CdtB muteins can be proposed as less immunogenic and stable therapeutic agents but the mutein 1 with a lower immunogenicity and a higher stability is preferred.

## 4. Conclusions

Most toxins such as the CdtB are restricted to apply as therapeutic agents due to having high immunogenicity, resulting in the production of anti-toxin antibodies. We succeeded in identifying the key residues affecting the immunogenicity of the CdtB. We also performed the best substitution (N19F, G74I, S161W) to find a stable mutein with less immune reactivity. However, computational approaches are functional and cost-effective methods to reduce the immunogenicity of the engineered therapeutic proteins and experimental studies are crucial to confirm its results.

## 5. Materials and Methods

**Figure 8:**
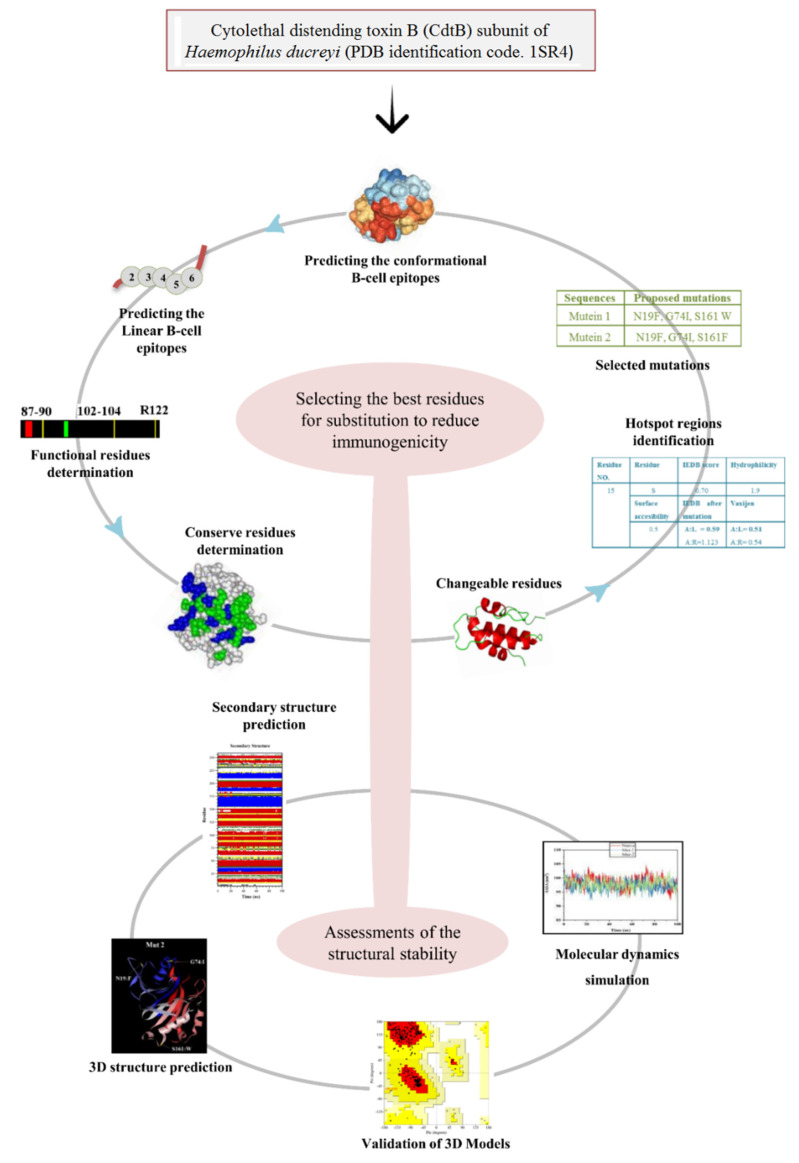
Flow charts represent methodology applied for designing the mutants with reduced immunogenicity by computational methods.

The methodologies used to reduce the antigenic residues of CdtB are shown in Figure 8. The servers employed in this study are shown in Table 6.

### 5.1. Sequence Analysis

The amino acid sequence of cytolethal distending toxin B (CdtB) subunits of *Haemophilus ducreyi* (UniProt accession number. O06523) were obtained from the UniProt tool (https://www.uniprot.org). The sequence was saved in the Fasta format, and the 3D structure of CdtB from PDB 1SR4 (https://www.rcsb.org/structure/1sr4) was stored for further studies.

### 5.2. Predicting Antigenicity

Antigen probability of CdtB was predicted by VaxiJen tool (www.ddg-pharmfac.net/vaxijen) [37].

### 5.3. Predicting the Linear B-Cell Epitopes

Several tools were used to predict the B-cell epitopes including BepiPred [52], ABCpred [41], SVMtrip [42], and BCPred [53]. BepiPred 2.0 (http://www.cbs.dtu.dk/services/BepiPred/) with threshold of 0.5 was used to predict the linear B-cell epitopes using the combination of hidden Markov method with one of the best propensity scale methods.

ABCpred (http://crdd.osdd.net/raghava/abcpred/) with a threshold of 0.83 was also employed to predict linear B-cell epitopes based on artificial neural networks (ANN). The tool is used for training of 700 B-cell epitopes and 700 non B-cell epitopes (random peptides) of maximum length of 20 residues.

Additionally, SVMTriP (http://sysbio.unl.edu/SVMTriP/) with a threshold of 0.8 and BCPred (http://crdd.osdd.net/raghava/bcepred/) with threshold of 1.0 were used to predict the linear B-cell epitopes. Both of these tools are based on support vector machines (SVM). However, SMVtrip is trained on length-fixed tripeptide composition vectors, while the BepiPred training is based on five string kernels that eliminate the need for representing the sequence into length-fixed feature vectors.

### 5.4. Predicting the Conformational B-Cell Epitopes

Several available tools were used to predict conformational B-cell epitopes. DiscoTope 2.0 (http://www.cbs.dtu.dk/services/DiscoTope/) [70] with a threshold of −3.7 was employed for detection of conformational B-cell epitopes. The tool is based on the calculation of surface accessibility and propensity amino acid score using 3D-structures of proteins. Subsequently, for further analysis of conformational B-cell epitopes, ElliPro (http://tools.iedb.org/ellipro/) which aims to identify geometrical structure properties was applied.

### 5.5. Predicting the Conseverd and Functional Residues

The relationship of the CdtB protein with the known proteins in the PDB, along with the similarity in their functionally important binding regions, were analyzed through ProBis tool (http://probis.cmm.ki.si/) by mapping structural similarity scores based on their physicochemical properties. Then, active site residues of the CdtB were determined according to the structural alignments with Z score > 2.0.

Moreover, PredictProtein tool (https://www.predictprotein.org/) was used to identify enzymatic sites that are crucial for the function of CdtB such as N-myristoylation, Casein kinase II phosphorylation, and Protein kinase C phosphorylation sites. Conserved region of CdtB was also determined by the Predict Protein tool. It is noteworthy that active sites and crucial functional sites, and highly conserved residues usually are excluded from mutagenesis analysis.

### 5.6. Predicting the Mutability Residues

I-Mutant2.0 (http://folding.biofold.org/i-mutant/i-mutant2.0.html) was used to predict stability changes upon every single point mutation of the determined residues of CdtB. The tool is a derivative of the ProTherm database that presents the most comprehensive available database of thermodynamic experimental data of free energy changes of protein stability upon mutation under different conditions. The condition for the protein mutation was performed under condition with pH 7.4 at the temperature of 37 °C, which conforms to human physiological conditions. If the Gibbs free energy difference was positive, the mutation is allowed to indicate increased protein stability. The free energy difference, ΔΔG, was calculated with the formula of ΔG (mutant protein) − ΔG (wild-type protein). The stabilizing alternate residues were replaced in the wild-type sequence. Then, the antigenic B-cell epitope scores of mutated residues were re-evaluated by the Immune Epitope Data Base and analysis resource (IEDB) (http://tools.iedb.org/bcell) and VaxiJen (http://www.ddgpharmfac.net/vaxijen/VaxiJen/VaxiJen.html) tools. In addition, surface accessibility, flexibility, and hydrophilicity scores of mutated residues were also acquired through the IEDB database.

### 5.7. Dstructure Prediction and Energy Minimization

For the existence of a close homologous sequence to the query, the homology modeling method was applied to 3D structure prediction at SWISS-MODEL (https://swissmodel.expasy.org/). SWISS-MODEL is used for automated comparative modeling of tertiary protein structures. The 3D modeled structure was refined using a 3D refine server at (http://sysbio.rnet.missouri.edu/3Drefine/). So, PDB of the initial 3D model taken from SWISS-MODEL was given to the 3Drefine server for energy minimization.

### 5.8. Validation of 3D Models

To evaluate the favored allowed or outlier region of 3D models, Ramachandran plot by PROCHECK program (http://servicesn.mbi.ucla.edu/PROCHECK/) was derived. The best 3D models were qualitatively assessed by Verify3D (http://services.mbi.ucla.edu/Verify_3D/) and ERRAT (http://services.mbi.ucla.edu/ERRAT/) program. Additionally, the ProSA-web at (https://prosa.services.came.sbg.ac.at/prosa.php) was applied to quantify z-score that indicates overall model quality.

### 5.9. Computational Methods

The MD simulations were performed using the GROMACS simulation package, version 5.1.4 [71,72]. With appropriate concentration of sodium and chloride ions all systems were neutralized. All the simulation systems were solvated in the SPC water model and periodic boundary conditions (PBC) were applied to the all simulation box axes [73]. The LINCS algorithm [74] was used to constrain all covalent bonds. The distance cut-off for the Van der Waals interactions and short-range electrostatic interactions were set to 1.2 nm in all simulations. The long-range electrostatic interactions were calculated using Particle Mesh Ewald (PME) algorithm. The energy-minimization and equilibration for all the systems were, respectively, performed through the steepest descent algorithm and NVT ensemble for 500 ps. Further equilibration was gradually provided for all the systems through the NPT ensemble. Constant pressure and temperature at 1 bar and 310 K were achieved through the Parrinello-Rahman barostat [75] and the Nose–Hoover algorithm thermostat. [75,76,77]. Each MD case was simulated for 100 ns with time steps of 2 fs.

## Figures and Tables

**Table 1:** Analysis of substitution mutation stability with difference in Gibbs’ free energy method by I-Mutant 2.0 Condition of mutation was set at pH of 7.4 and temperature of 37 °C. The residues with positive values of ΔΔG, were selected for further analysis. The residues with the lowest scores for Vaxijen, IEDB, hydrophilicity and Surface accessibility were excluded. Bold mutated residues have the best IEDEB and Vaxijen scores.

Residue NO.	Residue	Hydrophilicity	Surface Accessibility	IEDB Score (before Mutation)	IEDB Score (after Mutation)	Vaxijen
**16**	S	4.3	0.5	0.77	**S:V = 0.28** **S:L = 0.05** **S:I = 0.12** **S:M = 0.19** **S:F = 0.10** S:W = 0.35 S:A = 0.62 S:T = 0.73 S:H = 0.50 S:R = 0.57 S:K = 0.63 S:Q = 0.75 S:E = 0.82 S:D = 0.91	**S:V = 0.49** **S:L = 0.48** **S:I = 0.48** **S:M = 0.48** **S:F = 0.47** S:W = 0.47 S:A = 0.49 S:T = 0.50 S:H = 0.49 S:R = 0.50 S:K = 0.50 S:Q = 0.50 S:E = 0.50 S:D = 0.50
**17**	A	4.5	0.8	1.17	**A:L = 0.59** A:R = 1.123	**A:L = 0.51** A:R = 0.54
**19**	N	4.5	1.3	0.83	N:V = 0.34 **N:L = 0.11** N:I = 0.18 N:M = 0.26 **N:F = 0.16** N:W = 0.41 N:Y = 0.47 N:T = 0.8 N:C = 0.28 N:R = 0.64 N:E = 0.89 ND = 0.98	N:V =0.51 **N:L = 0.50** N:I = 0.51 N:M = 0.50 **N:F = 0.50** N:W = 0.49 N:Y = 0.50 N:T = 0.50 N:C = 0.49 N:R = 0.51 N:E = 0.50 ND = 0.50
**20**	E	2.2	1.3	0.71	E:V = 0.17 **E:L = −0.06** **E:I = 0.01** E:M = 0.08 **E:F = −0.009** **E:W = 0.24** E:T = 0.62 E:C = 0.1 E:R = 0.46 E:D = 0.80	E:V = 0.50 **E:L = 0.49** **E:I = 0.49** E:M = 0.49 **E:F = 0.49** **E:W = 0.47** E:T = 0.51 E:C = 0.49 E:R = 0.49 E:D = 0.49
**47**	S	3.0	0.5	1.23	S:V = 0.74 **S:L = 0.51** **S:I = 0.58**	S:V = 0.53 **S:L = 0.52** **S:I = 0.52**
**52**	L	3.2	0.8	0.62	A:V = 0.2 A:L = 0.05 **A:I = 0.1** **A:F = 0.1** A:W = 0.35 E A:P = 1.13 E A:S = 0.77 E A:C = 0.21 A:R = 0.58 E	A:V = 0.52 A:L = 0.51 **A:I = 0.51** **A:F = 0.50** A:W = 0.50 A:P = 0.52 A:S = 0.54 A:C = 0.54 A:R = 0.54
**74**	G	1.34	1.16	0.53	**G:I = 0.2**	**0.51**
**75**	T	3.3	1.4	0.50	**T:M = −0.01** T:F = −0.01 T:W = 0.13 T:Y = 0.19 **T:C = −0.002** **T:D = −0.70**	T:M = 0.54 T:F = 0.55 T:W = 0.54 **T:Y = 0.53** **T:C = 0.53** T:D = 0.51
**77**	S	4.9	4.3	0.7	S:V = 0.2 **S:L = −0.01** S:I = 0.05 S:M = 0.13 S:F = 0.03 S:W = 0.2 S:Y = 0.3 S:A = 0.5 E S:P = 1.04 E	S:V = 0.54 **S:L = 0.54** S:I = 0.54 S:M = 0.54 S:F = 0.54 S:W = 0.53 S:Y = 0.53 S:A = 0.53 S:P = 0.53
**160**	S	4.1	1.0	1.27	**S:V = 0.78** **S:L = 0.55** S:D = 1.42	**S:V = 0.48** **S:L = 0.49** S:D = 0.47
**161**	S	3.7	1.9	1.57	S:V = 1.08 **S:L = 0.85** **S:I = 0.92** **S:F = 0.90** **S:W = 1.15** S:D = 1.7	S:V = 0.47 **S:L = 0.46** **S:I = 0.46** **S:F = 0.45** **S:W = 0.45** S:D = 0.47
**162**	S	5.3	1.82	1.89	**S:V = 1.40 E** **S:L = 1.17 E** **S:I = 1.24 E** **S:F = 1.25 E** S:D = 2.03 E	**S:V = 0.53** **S:L = 0.52** **S:I = 0.52** **S:F = 0.52** S:D = 0.53

**Table 2:** Determination of hot spot B-cell epitope residues by VaxiJen and IEDB scores.

B Cell Epitopes (Native)	IEDB Score	VaxiJen	Mutation Positions	Alternative Amino Acids	IEDB Score	VaxiJen
LQGSSAV**N**ESKWNINVRQLLSGE (12–34)	1.9	0.88	19	F	0.78	0.31
L**G**TRSRPNM (73–81)	1.2	0.49	74	I	1.1	−0.31
S**S**SSPPERRVYS (160–171)	2.51	0.59	161	W F	1.9 1.7	0.16 0.25

**Table 3:** Immunogenic analysis by VaxiJen and IEDB scores of the muteins versus the native protein.

Sequences	IEDB Score	VaxiJen
Native	2.22	0.53
Mutein 1 (N19F, G74I, and S161F)	1.80	0.49
Mutein 2 (N19F, G74I, and S161W)	1.88	0.48

**Table 4:** Validation results were obtained by ERRAT and Verify3D online server.

Protein Structures	ERRAT *	Verify3D **
**Native**	95.635%	99.23%
**Mut 1**	86.905%	98.85%
**Mut 2**	90.873%	98.85%

* Good high-resolution structures generally produce values around 95% or higher. ** Percentage of the residues that had scores ≥ 0.2 in the 3D/1D profile.

**Table 5:** Mutein 1 and 2 have been compared in this table and their minimal differences have been listed.

Specifications	Mutein 1 (N19F, G74I, and S161F)	Mutein 2 (N19F, G74I, and S161W)
Ramachandran plot	89.7%	90.1%
RMSD profile	a steady state and a considerable thermostability through the simulation period	same pattern with the native model
RMSF factor	Less movements	more movements
The radius of gyration (Rg)	proper structural folding	proper structural folding
solvent- accessible surface area	The solvent accessible	The solvent accessible
Secondary structure	Stable β-sheet structures	Changes from alpha-helix to the coil and turn in some points

**Table 6:** Applied software and servers in this study are listed in order.

Server Name	Porpuse	Address
UniProt	Amino acid sequence	https://www.uniprot.org
PDB	3D structure	https://www.rcsb.org/structure/
VaxiJen tool	Antigen probability	www.ddg-pharmfac.net/vaxijen
BepiPred	Linear B-cell epitopes	http://www.cbs.dtu.dk/services/BepiPred/
ABCpred	Linear B-cell epitopes	http://crdd.osdd.net/raghava/abcpred/
SVMtrip	Linear B-cell epitopes	http://sysbio.unl.edu/SVMTriP/
BCPred	Linear B-cell epitopes	http://ailab-projects1.ist.psu.edu:8080/bcpred/
DiscoTope 2.0	Conformational B-cell epitopes	http://www.cbs.dtu.dk/services/DiscoTope/
ElliPro	Conformational B-cell epitopes	http://tools.iedb.org/ellipro/
ProBis tool	Functional residues	http://probis.cmm.ki.si/
PredictProtein tool	Identify enzymatic sites	https://www.predictprotein.org/
I-Mutant2.0	Mutability residues	http://folding.biofold.org/i-mutant/i-mutant2.0.html
SWISS-MODEL	3D structure prediction with homology modeling method	https://swissmodel.expasy.org/
PROCHECK program	Ramachandran plot	http://servicesn.mbi.ucla.edu/PROCHECK/
Verify3D	Validation of 3D Models	http://services.mbi.ucla.edu/Verify_3D/
ERRAT	Validation of 3D Models	http://services.mbi.ucla.edu/ERRAT/
ProSA-web	Overall model quality	https://prosa.services.came.sbg.ac.at/prosa.php
MD simulations	Thermodynamic stability	GROMACS simulation package, version 5.1.4
Immune Epitope Data Base and analysis resource (IEDB)	Antigenic B-cell epitope scores	http://tools.iedb.org/bcell

## Data Availability

Data sharing not applicable to this article as no datasets were generated or analysed during the current study.
